# Body image and mental health in university students: a scoping review of global evidence and research gaps

**DOI:** 10.3389/fpsyg.2026.1796613

**Published:** 2026-05-13

**Authors:** C. Pablos-Gabriel, Ana B. Sánchez-García, C. Patino-Alonso, J. C. Sánchez-García

**Affiliations:** 1 Social Service Affairs, Universidad de Salamanca, Salamanca, Spain; 2 Department of Didactic, Organization and Research Methods, Universidad de Salamanca, Salamanca, Spain; 3 Department of Statistics, Universidad de Salamanca, Salamanca, Spain; 4 Department of Social Psychology, Universidad de Salamanca, Salamanca, Spain

**Keywords:** body image, mental health, scoping review, self-esteem, suicidal ideation, university students

## Abstract

**Background:**

University students represent a high-risk population for psychological distress. While body dissatisfaction has emerged as a critical determinant of well-being, a comprehensive synthesis of contemporary evidence, mediating mechanisms, and systemic gaps is required to inform clinical practice.

**Objectives:**

To synthesize the scientific literature on body self-perception and mental health in university students, identifying core findings, mediating pathways, and existing research gaps.

**Methods:**

Following the PCC (Population, Concept, and Context) eligibility framework, a scoping review was conducted (March–April 2025), following the PRISMA-ScR guidelines. A systematic search was performed across Scopus, PubMed/MEDLINE, PsycINFO, SciELO, and Redalyc databases, including studies from 2014 to 2025 in English and Spanish. Eligibility criteria encompassed peer-reviewed quantitative, qualitative, and mixed-methods studies, as well as previous reviews; grey literature and unpublished theses were excluded. Two researchers performed independent data selection and extraction, followed by a narrative synthesis of the evidence. Full search strings are available as Supplementary material. In accordance with PRISMA-ScR guidelines for scoping reviews, formal quality appraisal of the included studies was not performed.

**Results:**

A total of 18 studies were included, which were categorized into four thematic clusters. Body dissatisfaction was consistently associated with elevated levels of depression and anxiety across diverse cultural contexts. Self-esteem and fear of negative evaluation were identified as primary mediators. Furthermore, dissatisfaction was significantly linked to suicidal ideation, impaired sleep quality, and social dysfunction. Methodologically, most studies utilized cross-sectional, quantitative designs and remained anchored in the gender binary, largely excluding gender-diverse populations.

**Conclusion:**

Body dissatisfaction is a potent risk factor for severe psychological distress in university students, driven by complex cognitive and digital mediators. There is an urgent need for longitudinal research and the inclusion of underrepresented populations. Findings highlight that Higher Education Institutions must transition toward proactive mental health frameworks, including integrated screening and inclusive, gender-affirming counseling policies.

**Systematic review registration:**

https://osf.io/6zefc.

## 1 Introduction

Body image, understood as the internal representation a person has of their own body, comprises perceptions, thoughts, and attitudes concerning physical appearance (Alipoor et al., 2009). In recent decades, concern about body image—especially body dissatisfaction—has emerged as a global public health issue (Bucchianeri and Neumark-Sztainer, 2014; Rodgers et al., 2023). This problem is exacerbated by contemporary sociocultural standards that promote ideals of extreme thinness or muscularity (McComb and Mills, 2022). This pervasive cultural expectation exerts a significant influence on the young university population, as the transition to higher education is accompanied by numerous stressors, including adapting to a new environment, achieving independence, and navigating a competitive social environment. These factors have the potential to exacerbate dissatisfaction with one’s physical appearance (Fitzsimmons-Craft, 2011; Huguenin et al., 2024).

University students are situated within *emerging adulthood*, a developmental stage marked by identity exploration and heightened sensitivity to social evaluation (Arnett, 2000, 2007). In this context, body image becomes a central component of self-concept, as increased social comparison and exposure to appearance-related norms, particularly within university and digital environments, may intensify body dissatisfaction and psychological vulnerability. In this context, body dissatisfaction may act as a psychological vulnerability factor that links developmental and sociocultural pressures to mental health outcomes in university students.

This phenomenon, in turn, has been shown to increase the risk of developing mental health problems such as anxiety, stress, and depression (García Barragán and Santos Pazos, 2025; Sheldon et al., 2021). Despite the growth in research in this field, the available evidence on the relationship between body image and mental health in university students is still fragmented and scattered.

On the one hand, from a conceptual perspective, the literature places body image within socio-cognitive frameworks that help to understand why body dissatisfaction is increasing among young people, particularly university students. The Tripartite Influence Model proposes that parents, peers, and the media act as sources of aesthetic pressure that operate through the internalisation of ideals (thinness/muscularity) and social comparison, proximal pathways that predict body dissatisfaction and psychological distress (Thompson et al., 1999; Keery et al., 2004; van den Berg et al., 2002). In parallel, Objectification Theory maintains that socialisation in contexts that objectify appearance promotes self-objectification, body surveillance and shame, processes linked to symptoms of anxiety and depression (Fredrickson and Roberts, 1997; Moradi and Huang, 2008). However, although these frameworks offer solid foundations, empirical evidence applied to the university context remains fragmentary, making it difficult to pinpoint how these processes operate in this population.

In terms of specific mechanisms, two routes are particularly relevant in today’s university life: (i) social comparison of appearance—common on social media—which increases dissatisfaction and negative affect (Fardouly et al., 2015; Holland and Tiggemann, 2016); and (ii) the internalisation of unrealistic aesthetic ideals, which mediates the relationship between exposure to highly visual content and psychological distress (Grabe et al., 2008; Rodgers et al., 2022). Recent meta-analyses confirm small to moderate but consistent associations between social media use and body image disturbance in young people, with more pronounced effects on highly visual platforms (Saiphoo and Vahedi, 2019; Rodgers et al., 2022). However, research on these dynamics within university populations seldom accounts for the measurement invariance of assessment instruments across sociodemographic variables—such as gender, sexual orientation, or weight status—thereby limiting the comparability of findings (Hazzard et al., 2022).

Despite the strength of these findings, there is still a need to systematically integrate the evidence on how these dynamics impact the mental health of university students.

Dissatisfaction with body image itself is a significant risk factor for the development of psychological distress (Liu B. et al., 2023) and is associated with harmful consequences for the mental health of young people worldwide (Merino et al., 2024). A substantial body of research has evidenced a correlation between body dissatisfaction and elevated rates of depression and anxiety, in addition to other psychological disorders (Barnes et al., 2020). This association has also been observed in non-Western populations, where body image satisfaction may operate as a protective factor for mental health in young women, as documented in a study with South African university students (Mwaba and Roman, 2009). A recent meta-analysis, for example, confirmed that body dissatisfaction is significantly correlated with symptoms of anxiety and depression, even in males (Barnes et al., 2020). In the case of males, concerns regarding muscularity (i.e., the drive for muscularity) are also pertinent, as they have been associated with depressive affect and anxiety (McCreary and Sasse, 2000; de Carvalho et al., 2019). The findings indicate that body image concerns extend beyond mere aesthetic issues, representing a pivotal aspect of young individuals’ mental well-being (Vincente-Benito and Ramírez-Durán, 2023). These relationships vary significantly according to sex, age, and socioeconomic status, as demonstrated by a comprehensive analysis with Spanish adolescents (Ramos et al., 2019). However, the extant literature on the subject is dispersed and not always differentiated by population group, which limits a comprehensive understanding of the phenomenon in university students.

Furthermore, the magnitude of these associations varies according to sociocultural and gender differences, though not invariably in the same direction. Female university students, for instance, tend to report greater dissatisfaction, but men are not immune to aesthetic pressure. In addition, the globalisation of body ideals coexists with cultural variations in the form that dissatisfaction and its mental health correlates take (Rodgers et al., 2020; Abdoli et al., 2024). Consequently, alongside the prevailing emphasis on the negative pole, the concept of positive body image is gaining traction. Conceptually distinct and associated with body appreciation/acceptance, embodiment, and a more benign reading of appearance information, its promotion is linked to greater well-being and constitutes a promising avenue for university interventions (Tylka and Wood-Barcalow, 2015; Wood-Barcalow et al., 2010; Burychka et al., 2021). However, the majority of studies address these factors in isolation, resulting in a paucity of integrated understanding of how they interact in the university experience.

Despite the growing body of research linking body image to mental health outcomes in university students, the existing evidence remains conceptually and methodologically fragmented. Studies differ widely in the variables examined, the mental health indicators considered, and the sociocultural contexts analyzed, limiting the integration of findings and their translation into effective university-based interventions. A scoping review is therefore warranted to systematically map the current evidence, identify key mechanisms and research gaps, and inform future research and prevention strategies in university settings.

The present scoping review has been undertaken to address the aforementioned issues by means of a comprehensive compilation and synthesis of the extant empirical literature, with a view to identifying the types of studies, their principal findings, and the extant knowledge gaps.

The objective of this study is twofold: firstly, to identify the main variables and relationships studied between body image and mental health in university students, and secondly, to detect areas that require further research to inform future interventions.

The subsequent review is thus guided by the following research questions:

- Which psychological (e.g., self-esteem, self-compassion) and sociocultural (e.g., social pressure, social media influence) variables have been identified as mediators or moderators in the relationship between body image and mental health among university students?
- What research methodologies predominate, and what are the main gaps and future research directions in the empirical literature examining body image and mental health among university students?

## 2 Materials and methods

### 2.1 Search strategy

A scoping review was conducted to explore the scientific literature on the relationship between body self-perception and mental health indicators in university students. This methodological approach was selected to map key concepts, identify gaps in research, and synthesise heterogeneous evidence. The methodology followed the guidelines of the PRISMA-ScR checklist (Tricco et al., 2018), ensuring transparency and rigour in the planning and reporting process.

The protocol for this scoping review was pre-registered on the Open Science Framework (OSF) on January 20, 2026 and will be made publicly available upon publication of this review at the following link: https://osf.io/r8297. Regarding the study timeline, an initial exploratory search was performed on April 21, 2025, to assess the volume of available literature and refine the research question. Crucially, the formal screening, full-text eligibility assessments, and data extraction were strictly conducted after the protocol registration in January 2026. This ensures that eligibility decisions were made according to the pre-defined criteria, blinded to the final synthesis results and followed.

Following this pre-registration, an updated systematic literature search was conducted in February 2026 across Scopus, PubMed/MEDLINE and PsycINFO databases. To ensure comprehensive coverage, Spanish-language databases and literature repositories such as SciELO and Redalyc were included, along with manual searches in specialised journals and Google Scholar to detect possible articles not indexed in the above databases. Specific search strategies were used for each database, combining key terms related to body image and mental health. This update confirmed that no additional studies published between April 2025 and February 2026 met the full eligibility criteria, thus maintaining the original selection of 18 studies.

The search was limited to publications from January 2014 to December 2025, and articles in English and Spanish were included. The full search strategy for each database, including specific keywords and Boolean operators, is provided in the Supplementary material (Appendix 1).

### 2.2 Inclusion and exclusion criteria

The eligibility criteria were defined using the PCC (Population, Concept, Context) eligibility framework, which is a standard approach for scoping reviews.

- Population: Studies focusing on university students (aged 18–30) were included, regardless of academic discipline or institutional characteristics.
- Concept: Studies examining the relationship between body self-perception (e.g., body dissatisfaction or weight concerns) and various mental health indicators (e.g., depression, anxiety, psychological well-being, eating disorders or related symptoms) were selected. A clear distinction was made between the conceptual boundary and the search strategy: while Eating Disorders (ED) were included as a conceptual eligibility criterion to capture the full spectrum of psychological distress, they were not used as primary search terms. This was a deliberate methodological choice to prioritize broader, non-clinical terms (e.g., “body concerns”) and ensure the inclusion of sub-clinical student populations, thereby avoiding a sample over-represented by clinical ED populations.
- Context: Studies had to have been published in English or Spanish between January 2014 and December 2025.

Articles exploring the correlation, mediating or moderating effects, or interventions in this population were included in the review. The review considered a wide range of methodological designs, including quantitative, qualitative and mixed-methods studies, as well as previous reviews. Studies that did not directly address the relationship between body image and mental health, as well as grey literature and unpublished theses, were excluded to ensure a level of evidence based on peer review. Two researchers performed data selection and extraction independently after January 20, 2026, and a third researcher resolved any discrepancies to ensure the reliability of the process.

### 2.3 Quality appraisal

While quality appraisal is often considered optional in scoping reviews, this study conducted a formal assessment to provide a more robust synthesis of the evidence. We employed the Joanna Briggs Institute (JBI) Critical Appraisal Tool for Analytical Cross-Sectional Studies (Moola et al., 2017) to evaluate the methodological quality of all included studies. Two researchers independently evaluated each study across eight specific domains, including the clarity of inclusion criteria, the validity and reliability of measurement instruments for exposure and outcomes, and the identification and management of confounding factors. Any discrepancies during the appraisal process were resolved through discussion and consensus with a third reviewer. The detailed results of this assessment are provided in the Supplementary Tables 3–5.

### 2.4 Data extraction and charting

Data extraction and charting were conducted by two independent researchers to ensure consistency and minimize potential bias. A standardized data charting form was developed and initially piloted using 20% of the included studies to refine categories and ensure comprehensive capture of relevant variables. The extracted data included: (i) study metadata (authors, year, country, and study design); (ii) participant demographics (sample size, age, and gender distribution); (iii) psychometric instruments utilized; (iv) primary outcomes regarding body image and mental health; and (v) identified mediators (e.g., self-esteem, social media influence) and research gaps. Discrepancies between reviewers were resolved through iterative discussion or consultation with a third expert reviewer until a 100% consensus was reached. This systematic approach ensured the reliability of the subsequent narrative synthesis.

Furthermore, due to the significant conceptual and methodological heterogeneity across the included studies—which involved diverse designs, psychometric instruments, and outcomes—a quantitative synthesis or meta-analysis was not feasible. Therefore, a narrative thematic synthesis was adopted to map and describe the evidence. To ensure the rigor of this process, the four thematic clusters were derived using an inductive thematic analysis approach. Two reviewers independently reviewed the findings extracted from each study to identify recurring patterns. This process involved: (i) initial coding of primary findings; (ii) axial coding to group related codes into broader categories based on conceptual similarities; and (iii) thematic consolidation into the final clusters presented in the results. Any discrepancies in categorization were resolved through iterative discussion until a 100% consensus was reached, ensuring the reproducibility of the synthesis.

In line with the exploratory nature of a scoping review, a formal assessment of the overall certainty of evidence (e.g., GRADE) was not performed. However, to enhance the transparency of our findings and address the methodological diversity of the included studies, a methodological quality appraisal of each individual study was conducted using the JBI Critical Appraisal Tool. This dual approach ensures that, while no global certainty rating is assigned to the body of evidence, the reliability of each specific result is underpinned by a rigorous independent evaluation and a double-blind verification process.

### 2.5 Summary of record selection

**Figure 1:**
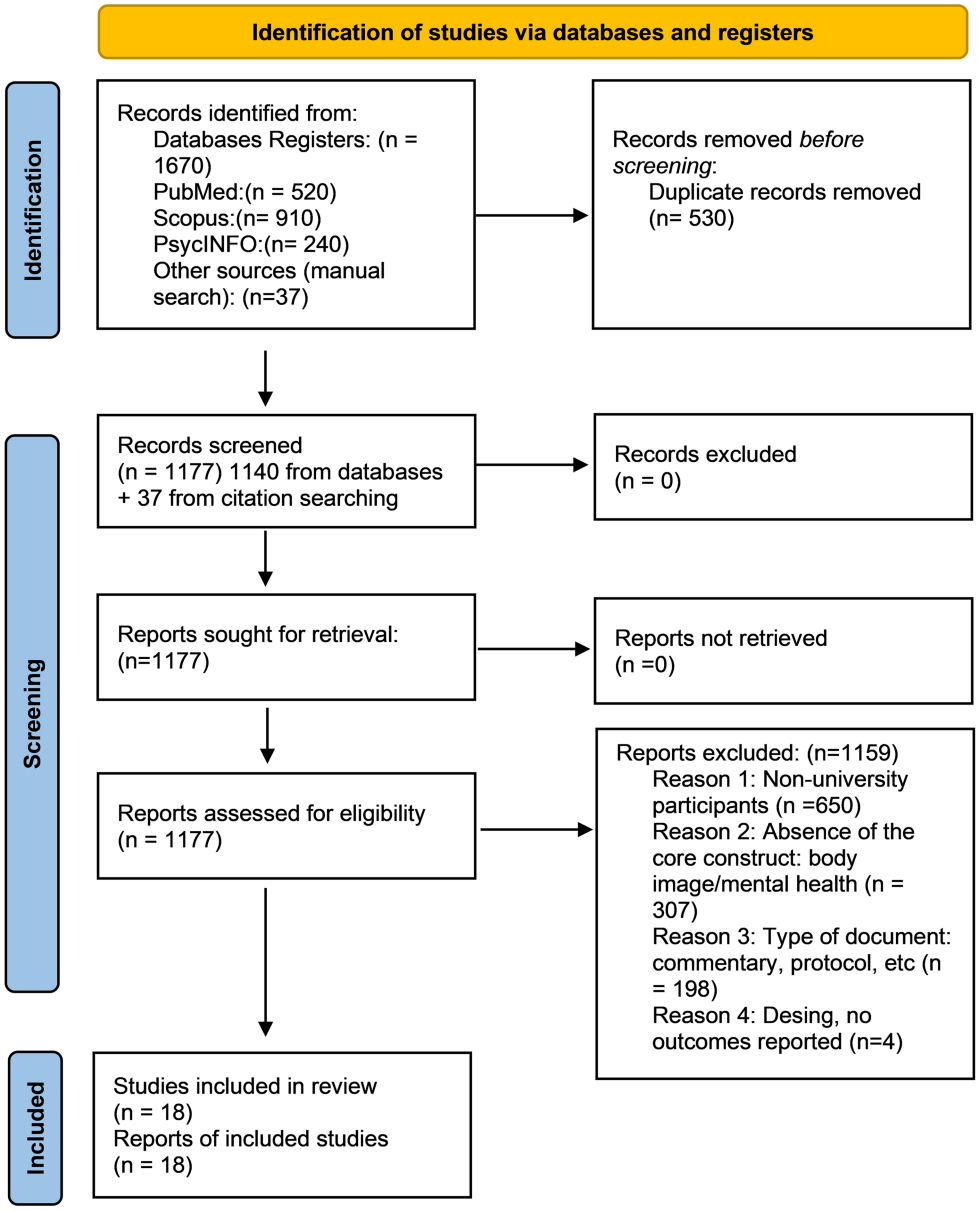
PRISMA 2020 flow diagram illustrating the study selection process. Adapted from Page et al. (2021) according to the PRISMA-ScR guidelines (Tricco et al., 2018). Distributed under the terms of the Creative Commons Attribution License (CC BY).

The systematic search across electronic databases yielded a total of 1,670 records: PubMed (*n* = 520), Scopus (*n* = 910), and PsycINFO (*n* = 240). After removing 530 duplicates using Mendeley, 1,140 unique records were identified for initial screening. To ensure a comprehensive evidence map, a manual backward search was performed by reviewing the reference lists of key primary studies and relevant systematic reviews. This strategy identified 37 additional eligible records. Consequently, a total of 1,177 records were prioritized for the title and abstract screening phase, which was conducted independently by two reviewers to minimize selection bias. The selection process is summarized in the PRISMA flow diagram (Figure 1), following the updated 2020 reporting guidelines (Page et al., 2021) adapted for the scoping review framework (Tricco et al., 2018). In accordance with PRISMA 2020 standards, a complete list of the studies excluded at the full-text review stage, along with the specific reasons for their exclusion, is provided in Supplementary Table 2 and available in the OSF repository.

## 3 Results

The methodological quality of the 18 included studies was rigorously evaluated using the JBI critical appraisal framework. Out of the total sample, 17 studies were formally scored, while one (Tylka and Wood-Barcalow, 2015) was retained as a foundational psychometric reference without a numerical score (N/A) to maintain methodological consistency. As shown in Supplementary Table 3, the evidence is characterized by a high degree of psychometric reliability but significant gaps in epidemiological control. Regarding the overall quality distribution:

- 23.5% (*n* = 4) of the appraised studies reached a High Quality rating. This group includes the qualitative exploration by Ogle et al. (2023), the randomized controlled trial by Smith et al. (2024), and the analytical studies by Chedid et al. (2025) and Edlund et al. (2022).
- 76.5% (*n* = 13) were classified as Moderate Quality.

### 3.1 The predominant “moderate” classification is primarily due to two systemic limitations

(1) The absence of explicit clinical exclusion criteria for pre-existing eating disorders (JBI Q1), and (2) the failure to adjust for Body Mass Index (BMI) as a primary confounder in multivariate models (JBI Q5 and Q6). Although the internal consistency of the instruments used was high across all studies, the lack of control for these physiological and clinical variables limits the ability to establish robust directional associations in the cross-sectional data. Characteristics of the sample of selected studies.

#### 3.1.1 Geographical coverage

The 18 studies cover various regions, with Asia (≈61%) predominating. The approximate distribution is: Asia 61% (11/18) —China, India, Pakistan, Saudi Arabia, Lebanon, Jordan—; Latin America 17% (3/18) —Mexico, Peru, Ecuador—; North America 17% (3/18) —US/Canada—; Europe 6% (1/18) —Sweden—; Africa 0% in this subset.

#### 3.1.2 Publication timeline

**Figure 2:**
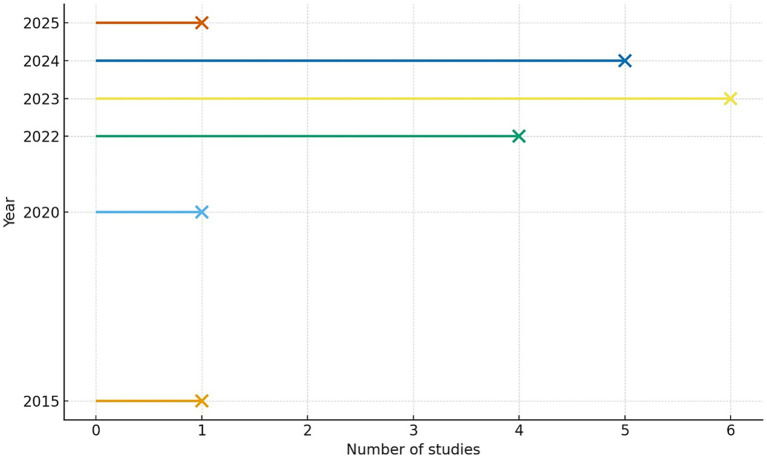
Temporal distribution of the included studies. Horizontal lollipop plot illustrating the number of studies published per year. The horizontal lines and markers indicate a significant increase in research productivity between 2022 and 2025, with a peak of 6 studies reached in 2023.

There has been a clear increase in recent publications. Overall, 83% of the studies were published between 2022 and 2024, with a peak in 2023, reflecting growing interest (see Figure 2).

#### 3.1.3 Sample size and language

18 studies report sample size (see characteristics in Table 1) with a combined total of ≈ 14,514 students. The typical size was medium (median ≈ 454; IQR ≈ 308–1,135). By range: 6 studies included >1,000 participants (Edlund et al., 2022; Hao et al., 2023; Liu B. et al., 2023; Palmeros-Exsome et al., 2022; Wang et al., 2023; Wu et al., 2023); 9 studies raged between 200 and 600 (Abbas et al., 2024; Abdulwahab et al., 2024; Chedid et al., 2025; Diengdoh and Ali, 2022; Estrada-Araoz et al., 2024; Hong and Ahmad, 2024; Jarrar et al., 2022; Mena-Freire et al., 2023; Sampath et al., 2020); and 3 included <300 (Abbas et al., 2024; Smith et al., 2024; Ogle et al., 2023). In terms of language, 15 articles are published in English and 3 in Spanish; most report sex distribution, showing a predominant representation of female students, with the exception of one all-male study (Sampath et al., 2020) and one focused on non-binary individuals (Ogle et al., 2023).

**Table 1:** Summary of characteristics and key findings of the included studies (*N* = 18).

Author (year)	*N*	Gender	Mean/(SD)/age range	Study design	Key variables and mediators	Main findings relevant to mental health
		Female (*n*, %)				
Abbas et al. (2024)	300	150	NR	Quantitative—cross-sectional	Individual body dissatisfaction (Hereafter IBD); GHQ-28 (depression, anxiety, social dysfunction, somatic symptoms)	Body dissatisfaction predicted worse scores on all subscales of the GHQ.
		50%	NR			
			18–35			
Abdulwahab et al. (2024)	392	NR	NR	Quantitative—cross-sectional	Social media use; body dissatisfaction (health science students, Saudi Arabia)	It reported a positive association between intensity of social media use and body dissatisfaction; a pattern consistent with international literature.
		63%	NR			
			18–25			
Chedid et al. (2025)	308	192	21.80	Quantitative—cross-sectional	Individual body dissatisfaction (IBD); self-esteem; suicidal ideation/behavior	IBD + low self-esteem predicted suicidal ideation/behavior. (outside the range 2014–2024; include it only if you decide to extend it)
		59.13%	± 4.07			
			18–35			
Diengdoh and Ali (2022)	358	NR	19.7	Quantitative—cross-sectional	Body satisfaction; depression; anxiety; self-esteem	↓ Self-esteem and ↑ anxiety predicted ↓ body satisfaction (*R*^2^ ≈ 0.15).
		56.1%	± 1.19			
			18–24			
Edlund et al. (2022)	4.263	2,645	25.1	Quantitative—cross-sectional (cohort study)	Individual body dissatisfaction; compulsive exercise; depression	IBD → ↑ depresión; el ejercicio compulsivo mostró asociación inversa con depresión.
		62%	± 6.4			IBD → ↑ depression; compulsive exercise showed an inverse association with depression.
			≥18			
Estrada-Araoz et al. (2024)	306	178	NR	Quantitative—correlational	Body dissatisfaction; eating disorder symptoms; anxiety	Significant correlations between body dissatisfaction, anxiety, and eating disorder symptoms.
		58.2%	NR			
			18–35			
Hao et al. (2023)	1.258	628	NR	Quantitative—cross-sectional	Body dissatisfaction; exercise; sleep quality; depression	↑ Body dissatisfaction and poorer sleep → ↑ depression; physical activity alleviated depression.
		49.9%	NR			
			18–23			
Hong and Ahmad (2024)	318	201	NR	Quantitative—cross-sectional	Body dissatisfaction; depression; anxiety; sex/course	↓ Body satisfaction → ↑ depression/anxiety; differences by course; consistent pattern in Chinese context.
		63.2%	NR			
			18–24			
Jarrar et al. (2022)	432	175	NR	Quantitative—mediation (cross-sectional)	Use of social media; social anxiety; body dissatisfaction	Social anxiety mediated the relationship between heavy social media use and body dissatisfaction.
		43%	NR			
			18–25			
Liu et al. (2023)	1.258	628	19	Quantitative—cross-sectional	Body dissatisfaction; sleep; exercise; depression	Poorer sleep quality, sedentary lifestyle, and body dissatisfaction → ↑ depression; women > men.
			± 1.03			
		49.9%	NR			
Mena-Freire et al. (2023)	476	283	21.24	Quantitative—SEM	Body image (latent); subjective well-being	Positive body image → ↑ well-being; indirect pathways via self-esteem; invariance by gender.
			± 3.65			
		61.5%	NR			
Ogle et al. (2023)	15	15	NR	Qualitative—interviews	Positive body image; wellbeing; coping	Topics: social comparison, stigma, coping strategies with an impact on well-being.
		Nonbinary	NR			
			≥18			
Palmeros-Exsome et al. (2022)	1.399	696	NR	Quantitative—cross-sectional	Body satisfaction/dissatisfaction; risky eating behaviors; nutritional status	Greater dissatisfaction and adiposity were associated with a higher risk of risky eating behaviors.
		50.5%	NR			
			18–29			
Sampath et al. (2020)	511	0	20.7	Quantitative—cross-sectional	IBD in males; sociocultural pressure; self-esteem	High IBD in males; sociocultural pressure and internalization of muscularity were relevant.
		0%	± 1.88			
			18–25			
Smith et al. (2024)	66	66	19.1	Quasi-experimental study	Social media break; self-esteem; body image	1 week break from social media → ↑ self-esteem and improved body image.
		100%	± 1.57			
			17–24			
Tylka and Wood-Barcalow (2015)	675	367	20.34	Psychometrics	Body appreciation (BAS-2)	Evidence of validity and reliability of the BAS-2 for adolescents/adults. Body appreciation predicts well-being beyond negative body image
			± 5.08			
			18–56			
Wang et al. (2023)	1,044	NR	19.34	Quantitative—cross-sectional (mediation model)	Body image ‘construction’; mental health; exercise	Body image was associated with mental health; exercise partially mediated the relationship.
			NR			
			NR			
Wu et al. (2023)	1,135	580	18.8	Quantitative—cross-sectional	Body dissatisfaction; depression; dietary habits	Greater body dissatisfaction and higher depression were associated with less healthy dietary habits among university students in southern China during COVID-19.
		NR	± 1			
			18–23			

*N* corresponds to the analytical sample reported by each study; when the size is not available, NR is indicated. To maintain methodological consistency, participants excluded by the primary authors due to incomplete data or failure to meet validation criteria were not included in the reported totals, prioritizing the sample size upon which the statistical findings were based. The classification of the design is determined by the original description. Variables/constructs is a comprehensive list of the central constructs of body image and mental health (or their correlates), and, when relevant, mediators/moderators. The findings of the study offer a concise summary of the primary result, or association, that has been documented. It is important to note that given the heterogeneity of the measures and instruments utilized, the effect sizes are not standardized or quantitatively compared across studies.

Regarding the study by Tylka and Wood-Barcalow (2015), data extraction was strictly limited to Study 1 (*N* = 675). This methodological choice was made to ensure population homogeneity, as Study 1 represents the primary derivation sample of college students (M age = 20.34; SD = 5.08), aligning with our inclusion criteria. Study 2 was excluded to avoid statistical redundancy, and Study 3 was omitted due to its community-based nature and significantly higher age mean, which falls outside the scope of this systematic review on emerging adulthood in academic settings.

#### 3.1.4 Studies design

Most studies used quantitative methods (14/18; ~78%), with one qualitative, one quasi-experimental and one psychometric study. All studies show a link between dissatisfaction/body image and psychological distress (depression, anxiety), as well as other correlates such as well-being, sleep quality and eating behaviors. Several studies point to relevant mechanisms (e.g., social media use, self-esteem and physical activity as mediators/modulators).

The analysed corpus shows a clear predominance of quantitative cross-sectional designs, with few qualitative and experiential/interventional approaches in the university population. Longitudinal research is practically non-existent; the only longitudinal study identified models trajectories of psychological distress but does not include body image as a central variable, which limits causal inferences. There is a high degree of heterogeneity in the instruments used, with little assessment of measurement invariance by gender or culture and irregular use of key covariates (time spent on social media, physical activity, sleep quality), which adds noise and potential bias. Geographically, there is a concentration in Asia and underrepresentation of specific European contexts and non-Hispanic Latin America, as well as samples with a female majority and few analyses by gender identity. In summary, there is a lack of prospective studies, controlled trials focused on improving body image with mental health outcomes, and mixed designs that integrate subjective experience with standardised psychometric indicators.

Among the included studies, certain psychometric validation works (e.g., Tylka and Wood-Barcalow, 2015) were retained not only for their methodological contribution to scale development but primarily for the robust correlational evidence they provide regarding the links between body appreciation and psychological well-being in the university context.

### 3.2 Qualitative findings

Based on the narrative synthesis of the included studies, the findings were grouped into four overarching themes describing the relationship between body image and mental health in university students.

#### 3.2.1 Body dissatisfaction and depression/anxiety

Review of literature reveal a small-to-moderate link between body image dissatisfaction and symptoms of depression/anxiety in university students (e.g., Hao et al., 2023; Liu X. et al., 2023; Liu B. et al., 2023; Hong and Ahmad, 2024). This link is consistent across different sociocultural contexts, underscoring its global relevance.

In Asia, lower body satisfaction is linked to more depression/anxiety in China (Hong and Ahmad, 2024), and there are variations by academic year. In India, research showed that more anxiety/lower self-esteem predicts lower body satisfaction (Sampath et al., 2020; Diengdoh and Ali, 2022).

In Latin America, body dissatisfaction is linked to psychological distress and concurrent problems. In Peru, there are significant associations between dissatisfaction, ED symptoms and anxiety (Estrada-Araoz et al., 2024). In Mexico, Palmeros-Exsome et al. (2022) linked greater dissatisfaction/adiposity to risky eating behaviors.

The relationship with depression is equally robust. In a cross-sectional cohort from Sweden, Edlund et al. (2022) observed that body dissatisfaction is associated with more depressive symptoms. In China, elevated levels of dissatisfaction, in conjunction with suboptimal sleep and sedentary behavior, were associated with increased depression scores (Hao et al., 2023; Liu B. et al., 2023). This association remained consistent in the context of the ongoing pandemic.

A salient nuance pertains to the role of exercise. Whilst there is a body of literature positing that dissatisfaction can engender compulsive exercise behaviors, Edlund et al. (2022) found an inverse association between compulsive exercise and depression, thus suggesting the possibility of temporary mood-lifting effects. The prevailing consensus indicates that body dissatisfaction functions as a risk factor for depression and anxiety, a phenomenon likely mediated by negative self-evaluation and self-criticism for the modulating role of exercise (see Wang et al., 2023).

#### 3.2.2 Impact on well-being, self-esteem and other mental health issues

The association between body dissatisfaction and a myriad of psychological consequences extends beyond depression and anxiety. The following is a list of the most salient consequences:

##### 3.2.2.1 Self-esteem and suicide risk

Self-esteem is a key factor. In Chedid et al. (2025) found that body dissatisfaction and low self-esteem predict suicidal ideation/behavior in university students. The proposed model suggests that body dissatisfaction leads to decreased self-esteem, which in turn increases the risk of hopelessness and suicidal behavior (Chedid et al., 2025). This finding is alarming, as it links body image to serious consequences beyond the usual emotional distress.

##### 3.2.2.2 Psychological well-being and daily functioning

Despite the absence of evidence demonstrating a significant relationship between well-being and psychological well-being, the prevailing trend indicates that a positive body image is associated with greater subjective well-being (Mena-Freire et al., 2023) and that the cultivation of positive resources notably body appreciation is a promising area of research (Tylka and Wood-Barcalow, 2015). In addition, recent literature has identified a correlation between dissatisfaction with sleep quality and greater discomfort: substandard sleep and reduced physical activity are concomitant with elevated levels of depression (Hao et al., 2023; Liu B. et al., 2023). In the domain of social functioning, research employing global mental health indicators (GHQ) has demonstrated a correlation with social dysfunction (Abbas et al., 2024).

##### 3.2.2.3 The relationship with risky eating behaviors

Body dissatisfaction constitutes a pivotal component of eating disorders (EDs) and is associated with extreme dieting, binge eating and less healthy eating habits. Correlations have been discovered between dissatisfaction, ED symptoms, and anxiety in Peru (Estrada-Araoz et al., 2024), while in China, greater body dissatisfaction and depression are associated with poorer dietary habits during the pandemic (Wu et al., 2023). Furthermore, Palmeros-Exsome et al. (2022) posit that adiposity and dissatisfaction are associated with risky eating behaviors in Mexican university students.

#### 3.2.3 Gender, sociocultural context and other factors

The literature identifies several modulating factors in the relationship between body self-perception and mental health, including gender, sociocultural context and other individual elements.

Female university students report higher levels of body dissatisfaction and a stronger association with depression and anxiety. Men are not immune to these pressures, particularly concerns about muscularity and body definition, which is linked to poorer emotional adjustment, lower self-esteem, and greater psychological distress (Sampath et al., 2020). These differences suggest that, although the direction of the effect is similar in both sexes, the content of body concern and the normative criteria to which it responds are partially different.

The sociocultural context also shapes how dissatisfaction is expressed and relates to mental health. In Middle Eastern countries, intensive use of social media and social anxiety emerge as particularly relevant factors: in a multi-country sample, social anxiety mediated the link between social media use and body dissatisfaction, indicating that exposure to appearance-related content can trigger fears of social judgement and, with it, a deterioration in well-being (Jarrar et al., 2022). Convergently, a positive association between intensity of social media use and body dissatisfaction was observed in health science students in Saudi Arabia, a pattern consistent with the international literature and pointing to the role of digital environments in the homogenisation of aesthetic ideals (Abdulwahab et al., 2024).

In addition to gender and culture, other situational and developmental factors appear to modulate the effects. For instance, the academic stage and variations in body satisfaction, as well as psychological distress. The findings, drawn from a Chinese sample, reveal significant disparities based on the year of study. These observations suggest that periods of transition, such as the commencement or conclusion of a degree programme, may serve as catalysts for heightened social comparison and self-criticism concerning physical appearance (Hong and Ahmad, 2024). Furthermore, an association has been demonstrated between higher levels of physical activity and reduced symptoms of depression and anxiety in university students (Hao et al., 2023; Wang et al., 2023). This finding suggests that physical activity may offer a partial mitigation of the impact of body dissatisfaction on mood.

#### 3.2.4 The role of mediators and moderators in the context of the relationship between body image and mental health

Recent evidence delineates a series of psychosocial mechanisms that elucidate the association between body dissatisfaction and compromised mental health. Firstly, the hypothesis that self-esteem functions as a robust mediator is confirmed: the negative self-evaluation and self-criticism that accompany body dissatisfaction erode self-esteem, and, when this is low, the risk of affective and behavioral problems increases. In the Lebanese university population, the combination of dissatisfaction and low self-esteem predicts suicidal ideation and behavior (Chedid et al., 2025). Secondly, social anxiety and appearance comparison linked to social media use are a key explanatory pathway: greater exposure to appearance-related content increases unfavourable comparison and raises social anxiety, which in turn mediates the link with dissatisfaction and distress (Jarrar et al., 2022).

A third set of mechanisms is attributable to lifestyle. During the pandemic, studies have demonstrated that insufficient sleep, sedentary lifestyles, and body dissatisfaction have co-occurred and been associated with increased depression among university students. This triad of factors has been shown to act as a perpetuating cycle of distress (Hao et al., 2023; Liu B. et al., 2023). Conversely, the presence of positive resources, notably regular physical activity, has been demonstrated to be associated with enhanced mental well-being. These positive resources have been shown to act as a mitigating factor in the impact of dissatisfaction on symptoms, thereby establishing a link within the explanatory chain (Wang et al., 2023).

With regard to the role of gender in moderation, studies have shown that the effects tend to intensify the strength of associations in women, although the effects remain present in men, where body concerns are more oriented towards muscularity (Sampath et al., 2020). The relationship is also influenced by body composition/BMI: stronger associations between dissatisfaction and risky eating behaviors are observed in contexts with higher adiposity, probably due to a combination of internal pressure and external labelling (Palmeros-Exsome et al., 2022).

In summary, the influence of body dissatisfaction on mental health is neither linear nor one-dimensional; rather, it operates through networks of mediation, including self-esteem, social anxiety, sleep and physical activity. This influence is modulated by factors such as gender and BMI. This underscores the necessity for multifaceted interventions that, within the university environment, encompass the integration of body acceptance and appreciation, the mitigation of social comparison (particularly on social media platforms), and the promotion of physical activity and the adoption of healthy sleep habits.

## 4 Discussion

The findings of this scoping review suggest a robust association between body image dissatisfaction and psychological distress within the university population. The evidence reviewed indicates a relationship between body dissatisfaction and symptoms of depression and anxiety, thereby supporting the notion that body self-perception is a central component of self-concept. This work contributes to the existing body of knowledge by indicating that body dissatisfaction may function as a potencial risk factor for a broader range of psychological problems, including but not limited to low self-esteem, stress, suicidal ideation, and social dysfunction.

It is particularly salient that this pattern is observed across different cultures and contexts. The influence of global media and social networks has been identified as a contributing factor to the homogenisation of body ideals, potencially generating similar psychological effects on a global scale (Rodgers et al., 2023). A comparison of these findings with those of previous systematic reviews (e.g., García Barragán and Santos Pazos, 2025) reveals a strong consistency, thereby reaffirming the observed links between body dissatisfaction and depression, anxiety, and social dysfunction. This scoping review also provides a complementary dimension by identifying mediators such as self-esteem and fear of negative evaluation, which support theoretical models such as Cash’s cognitive-behavioral model (Cash, 2012) in explaining potential pathways between the internalisation of beauty ideals and psychopathology.

These findings are consistent with sociocognitive frameworks such as the Tripartite Influence Model, which posits that sociocultural pressures, appearance-based comparison, and the internalization of aesthetic ideals play a central role in the development of body dissatisfaction and subsequent psychological distress (Thompson et al., 1999). The identification of mediating mechanisms, including self-esteem and fear of negative evaluation, further supports this model by illustrating how external appearance norms may be internalized and translated into negative self-evaluative processes among university students (Moradi and Huang, 2008). However, these theoretical advancements must be interpreted with caution in light of the methodological quality of the primary sources. The systematic evaluation of the 18 studies exposes a significant methodological stagnation in the field. Although recent research has transitioned toward sophisticated statistical modeling—such as Structural Equation Modeling (SEM) and complex mediation—the foundational data often remain “hollow” regarding physical health markers. The fact that over 76.5% of the evidence falls into the moderate quality category points to a systemic omission of the biological reality of the participants.

This “BMI gap” is not merely a formal deficit; it compromises the internal validity of the reported correlations. Without adjusting for objective weight status or clinical history, it is difficult to discern whether body dissatisfaction stems from purely psychological and sociocultural constructs or if it is significantly anchored in unmeasured physiological factors. Future research in university settings must move beyond the “survey-only” paradigm and adopt multimodal designs that treat BMI and clinical health records as non-negotiable variables rather than optional data points.

## 5 Conclusions, limitations, and implications

From a theoretical standpoint, the findings suggest a reinforcement of existing models in the field, with body dissatisfaction highlighting as a factor of psychological vulnerability in emerging adulthood. The identification of mediators has the potential to enrich cognitive and social theories by providing empirical evidence on the possible pathways linking body perception to mental health.

From a pragmatic perspective, our findings underscore the necessity for academic institutions to transition from awareness to action. It is no longer sufficient to merely acknowledge body image as a peripheral concern; university mental health services must adopt comprehensive promotion strategies that integrate this dimension into routine assessment and support protocols. Crucially, the implementation of such strategies is supported by a growing body of evidence-based interventions. Acceptance-based behavioral models have demonstrated significant efficacy in reducing body-related distress (Margolis and Orsillo, 2016), while dissonance-based programs, such as the *Body Project*, remain a gold standard for mitigating eating disorder risk (Griffen et al., 2018; Stice et al., 2018).

Furthermore, the emergence of digital and mindfulness-based protocols offers scalable and cost-effective solutions tailored to the university environment (Dover and Clements, 2025; Liu et al., 2025). By incorporating programs that foster body appreciation, self-compassion, and resilience, institutions can bridge the gap between clinical evidence and campus-wide wellbeing. The challenge for future initiatives lies not in the lack of effective tools, but in the systematic integration of these validated interventions into the institutional fabric of higher education.

While this scoping review provides a comprehensive mapping of the current literature, future research can build upon these findings by addressing certain methodological considerations. The primary objective was to map the breadth and nature of the evidence rather than to provide a critically appraised or aggregated synthesis; therefore, the findings should be interpreted with this methodological boundary in mind. Furthermore, it is important to acknowledge a transparency limitation regarding the study’s timeline. While the formal protocol was registered on the Open Science Framework (OSF) in January 2026, an initial exploratory search was conducted in April 2025 to assess the volume of literature and refine the research question. Although the systematic screening, eligibility decisions, and data extraction were strictly performed following the pre-registered protocol after January 2026, ensuring that the final synthesis remained independent of the initial exploratory pase.

Beyond this, the interpretation of the synthesized evidence must account for the predominantly cross-sectional nature and the reliance on self-report methodology of the primary studies. These characteristics limit the ability to establish causality and may introduce common-method bias; consequently, the reported associations are presented as suggested correlates rather than definitive causal links. Additionally, the quantification of association magnitudes remains an important avenue to establish the strength of the evidence and effect sizes. This approach would significantly enhance the ability to weigh the robustness of the findings. Furthermore, the potential for reporting bias within the synthesized evidence must be considered. Since this review predominantly includes peer-reviewed, published literature, there is a risk of publication bias, where statistically significant results are more likely to be available than null findings. Consequently, the reported strength and prevalence of the associations between body image and mental health may be slightly overestimated. While this mapping provides a comprehensive overview of the field, readers should interpret the consistency of these associations acknowledging the potential underrepresentation of non-significant data. Beyond these considerations, while the nexus between body perception and mental health is statistically robust across all 18 studies, its clinical interpretation is limited by a lack of control over physiological confounders. Transitioning from moderate to high-quality evidence will require a more rigorous integration of physical health markers and stricter clinical exclusion criteria to move beyond descriptive correlations toward true predictive models. Consequently, the systematic process of this review has illuminated critical gaps in the current knowledge base, which represent priority areas for future research aimed at generating high-quality evidence, such as:

(i) Causality. The paucity of longitudinal studies makes it difficult to make definitive inferences regarding the direction of causality. It is imperative that prospective research is conducted in order to elucidate whether body dissatisfaction leads to psychopathology or whether, conversely, pre-existing psychopathology distorts body perception.
(ii) Interventions: A paucity of controlled trials (CTs) exists that evaluate whether enhancing body image is associated with improvements in mental health. This critical gap is a significant hindrance to the development of evidence-based interventions.
(iii) Underrepresented Populations: The majority of studies concentrate on the binary gender division, thereby creating a lacuna in research on the relationship in transgender or non-binary students, a population which is characterised by elevated levels of mental health vulnerability.
(iv) Mixed Methods: The preponderance of quantitative methodologies within extant literature signifies a necessity for qualitative or mixed-methodological studies, with a view to capturing subjective experiences and contextual nuances.
(v) Specific outcomes: The extant literature on the subject is not yet conclusive with regard to the impact of body dissatisfaction on less explored outcomes, such as academic performance, social functioning, or psychosomatic symptoms.

Drawing from this scoping review’s synthesis, it is suggested that Higher Education Institutions (HEIs) transition from traditionally reactive models toward proactive, systemic mental health frameworks. Central to this evolution is the implementation of integrated biopsychosocial screening protocols within university health services, enabling the early detection of body-related distress before it manifests as severe psychological morbidity. Furthermore, HEIs should prioritize critical media literacy interventions designed to dismantle the cognitive mechanisms of social comparison and the internalization of idealized body standards identified in the literature. Finally, psychological support services must adopt an intersectional and inclusive lens, integrating body-neutrality and gender-affirming practices to effectively address the unique stressors of students across diverse body types and gender identities. By embedding these evidence-based strategies, universities can foster a more resilient academic environment that acknowledges the complex interplay between physical self-perception and psychological well-being. Ultimately, as suggested in this scoping review, dissatisfaction with body image appears to be a significant correlate of mental health in young university students; therefore, addressing this issue necessitates the undertaking of research that is both rigorous and diversified, encompassing the analysis of mediators and the evaluation of interventions to advance clinical practice and institutional policy.

## Data Availability

The datasets presented in this study can be found in online repositories. The protocol and the planned data extraction framework for this scoping review are pre-registered on the Open Science Framework (OSF). Due to the ongoing nature of the research, the registration is currently under embargo. Upon publication, all relevant data and the full protocol will be made publicly available at https://osf.io/6zefc. Further inquiries can be directed to the corresponding author.
