# Supplementary material for: Body image and mental health in university students: a scoping review of global evidence and research gaps
Source: Front Psychol. 2026 May 13;17:1796613. doi: 10.3389/fpsyg.2026.1796613 (PMC13212270; doi:10.3389/fpsyg.2026.1796613)
Supplement: Supplementary file 1 [file Supplementary_file_1.pdf]

frontiers

Supplementary Material

1 Supplementary Data Sheet 1 | Full search strategies for all databases (Appendix 1).

The search will be conducted in PubMed/MEDLINE, Scopus, PsycINFO, SciELO, and Redalyc. The following search string (adapted for each database) will be used:

PubMed

English search: ("body image"[tiab] OR "body dissatisfaction"[tiab]) AND ("mental health"[tiab] OR depression[tiab] OR anxiety[tiab]) AND ("college students"[tiab] OR "university students"[tiab]). Limiters: publications 2014–2025; English.

Spanish search: ("imagen corporal"[tiab] OR "insatisfacción corporal"[tiab]) AND ("salud mental"[tiab] OR depresión[tiab] OR ansiedad[tiab]) AND ("estudiantes universitarios"[tiab] OR "estudiantes universitarias"[tiab]). Límites: publicaciones 2014–2025; idioma español.

Scopus

English search: TITLE-ABS-KEY (("body image" OR "body dissatisfaction") AND ("mental health" OR depression OR anxiety) AND ("college student" OR "university student")) AND PUBYEAR > 2013 AND PUBYEAR < 2026.

Spanish search: TITLE-ABS-KEY (("imagen corporal" OR "insatisfacción corporal") AND ("salud mental" OR depresión OR ansiedad) AND ("estudiantes universitarios" OR "estudiantes universitarias")) AND PUBYEAR > 2013 AND PUBYEAR < 2026.

PsycINFO

English search: ("body image" OR "body dissatisfaction") AND ("mental health" OR depression OR anxiety) AND ("college students" OR "university students"). Limiters: 2014–2025, English.

Spanish search: ("imagen corporal" OR "insatisfacción corporal") AND ("salud mental" OR depresión OR ansiedad) AND ("estudiantes universitarios" OR "estudiantes universitarias"). Límitadores: 2014–2025, español.

SciELO

English search: ("body image" OR "body dissatisfaction") AND ("mental health" OR depression OR anxiety) AND ("college students" OR "university students"). Limiters: 2014–2025.

Spanish search: ("imagen corporal" OR "insatisfacción corporal") AND ("salud mental" OR depresión OR ansiedad) AND ("estudiantes universitarios" OR "estudiantes universitarias"). Limitadores: 2014–2025.

Redalyc

English search: ("body image" OR "body dissatisfaction") AND ("mental health" OR depression OR anxiety) AND ("college students" OR "university students"). Limiters: 2014–2025.

Spanish search: ("imagen corporal" OR "insatisfacción corporal") AND ("salud mental" OR depresión OR ansiedad) AND ("estudiantes universitarios" OR "estudiantes universitarias"). Limitadores: 2014–2025.

Google Scholar

English search: ("body image" OR "body dissatisfaction") AND ("mental health" OR depression OR anxiety) AND ("college students" OR "university students") after:2013 before:2026.

Spanish search: ("imagen corporal" OR "insatisfacción corporal") AND ("salud mental" OR depresión OR ansiedad) AND ("estudiantes universitarios" OR "estudiantes universitarias") after:2013 before:2026.

2 Supplementary Data Sheet 2 | List of excluded studies (after full-text screening) (Appendix 2)

The following Tabla 2, details the studies retrieved for full-text assessment but subsequently excluded following the eligibility screening phase. To ensure methodological transparency and reproducibility, specific rationales for exclusion are provided for each record.

Table 2. List of excluded studies (after full-text screening)

**Full Citation**	**Primary Reason for Exclusion**
Casanova-Garrigós, G., et al. (2025). Influencia de las redes sociales en la imagen corporal de adolescentes: una revisión integrativa. *Enfermería Global, 24*(75).	*Wrong Population & Design*: The study focuses on adolescents (under 18) rather than university students and utilizes an integrative review design instead of primary data analysis.
Diengdoh, I., & Ali, A. (2022). Body Image and Its Association with Depression, Anxiety, and Self-esteem among College going Students. *Indian Journal of Community Medicine, 47*(2), 218-222. https://dx.doi.org/10.4103/ijcm.ijcm_881_21	*Conceptual Incongruence*: Not assessing both body image and mental health constructs together.
Duno, M., & Acosta, E. (2019). Percepción de la imagen corporal en adolescentes universitarios. *Revista Chilena de Nutrición,46*(5), 545-553. http://dx.doi.org/10.4067/S0717-75182019000500545	*Wrong Population*: The sample consisted of adolescents (developmental stage outside the 18–30 university student range defined in the PCC framework).
Fairburn, C. G., et al. (2003). Cognitive behaviour therapy for eating disorders: a "transdiagnostic" theory and treatment. *Behaviour Research and Therapy*, 41(5), 509-528. https://doi.org/10.1016/S0005-7967(02)00088-8	*Outside Timeframe & Clinical Focus:* The study was published prior to 2014 and focused exclusively on clinical populations with diagnosed eating disorders rather than the general university population.
Opoku, A. E. (2024). The Influence of Self Esteem and Body Image on the Mental Wellbeing of University of Ghana Students. *Annals Of Psychiatry And Treatment, 8*(1), 33-42. https://doi.org/10.17352/apt.000062	*Wrong Population:* Despite the title mentioning university students, the internal analysis focused on adolescent-specific developmental markers not applicable to the 18–30 university cohort.

3 Supplementary Data Sheet 3 | Risk of Bias Assessment (JBI Critical Appraisal) (Appendix 3)

This section presents the risk-of-bias assessment for the 18 studies included in this scoping review. To ensure methodological alignment, evaluations were conducted using three specialized Joanna Briggs Institute (JBI) Critical Appraisal Tools: The Checklist for Analytical Cross-Sectional Studies (n=15), the Checklist for Qualitative Research (n=1), and the Checklist for Randomized Controlled Trials (n=1). One psychometric validation study was retained for its foundational evidence but was not formally scored to avoid methodological mismatch (see Note *3). Each study was independently screened across specific domains, including sample representativeness, measurement reliability, and the management of confounding variables—particularly Body Mass Index (BMI) and clinical history. The results indicate that 23.5% (n=4) of the appraised studies achieved a High-Quality rating, while 76.5% (n=13) were classified as Moderate Quality. This distribution primarily reflects a systemic lack of multivariate control for physiological confounders in current cross-sectional literature.

Table 3. Risk of Bias Assessment (JBI Critical Appraisal)

Study	Q1	Q2	Q3	Q4	Q5	Q6	Q7	Q8	Score	Quality Rating
Abbas et al. (2024)	Y	Y	Y	Y	N	N	Y	Y	6/8	Moderate
Abdulwahab et al. (2024)	N	Y	Y	Y	N	N	Y	Y	5/8	Moderate
Chedid et al. (2025)	Y	Y	Y	Y	Y	Y	Y	Y	8/8	High
Diengdoh & Ali (2022)	Y	Y	Y	Y	N	N	Y	Y	6/8	Moderate
Edlund et al. (2022)	Y	Y	Y	Y	Y	Y	Y	Y	8/8	High
Estrada-Aráoz et al. (2024)	N	Y	Y	Y	N	N	Y	Y	5/8	Moderate
Hao et al. (2023)	N	Y	Y	Y	N	N	Y	Y	5/8	Moderate
Hong & Ahmad (2024)	N	Y	Y	Y	N	N	Y	Y	5/8	Moderate
Jarrar et al. (2022)	N	Y	Y	Y	N	N	Y	Y	5/8	Moderate
Liu et al. (2023)	N	Y	Y	Y	N	N	Y	Y	5/8	Moderate
Mena-Freire et al. (2023)	N	Y	Y	Y	N	N	Y	Y	5/8	Moderate
Ogle et al. (2023) *[1]	-	-	-	-	-	-	-	-	-	High
Palmeros-Exsome et al. (2022)	N	Y	Y	Y	Y	N	Y	Y	6/8	Moderate
Sampath et al. (2020)	N	Y	Y	Y	N	N	Y	Y	5/8	Moderate
Smith et al. (2024) *[2]	-	-	-	-	-	-	-	-	-	High
Tylka & Wood-Barcalow (2015) *[3]	-	-	-	-	-	-	-	-	-	NA
Wang et al. (2023)	N	Y	Y	Y	N	N	Y	Y	5/8	Moderate
Wu et al. (2023)	N	Y	Y	Y	Y	N	Y	Y	6/8	Moderate

Note: Y: Yes; N: No; NA: Not Applicable; NR: Not Reported. JBI Critical Appraisal Checklist for Analytical Cross-Sectional Studies: Q1: Were the criteria for inclusion in the sample clearly defined?/ Q2: Were the study subjects and the setting described in detail?/ Q3: Was the exposure measured in a valid and reliable way?/ Q4: Were objective, standard criteria used for measurement of the condition?/ Q5: Were confounding factors identified?/ Q6: Were strategies to deal with

confounding factors stated?/ Q7: Were the outcomes measured in a valid and reliable way?/ Q8: Was appropriate statistical analysis used?
*[1]Note: see Table 4.
*[2]Note: see Table 5.
*[3]Note: This study was included primarily for its robust empirical evidence regarding the associations between body appreciation and psychological well-being. Although categorized as a psychometric validation study, the correlational data from Study 1 (N = 675) met all eligibility criteria for this scoping review. To ensure population homogeneity and avoid redundancy, only data from the primary university-based sample (Study 1) were extracted. Given its foundational nature in scale development, this study was not subjected to the JBI Analytical Cross-Sectional Checklist to prevent methodological bias, as its primary design focused on instrument refinement rather than epidemiological risk-factor analysis.

Table 4. Quality Assessment of Qualitative Evidence (JBI Critical Appraisal Checklist)

**Study**	**Q1**	**Q2**	**Q3**	**Q4**	**Q5**	**Q6**	**Q7**	**Q8**	**Q9**	**Q10**	**Score**	**Quality Rating**
Ogle et al. (2023)	Y	Y	Y	Y	Y	N	N	Y	Y	Y	8/10	High

Note: JBI Critical Appraisal Checklist for Quality Assesment of Qualitative Evidence: Q1: Congruity between philosophical perspective and methodology /Q2: Congruity between methodology and research question /Q3: Congruity between methodology and data collection methods/ Q4: Congruity between methodology and representation/analysis of data/Q5: Congruity between methodology and interpretation of results/ Q6: Statement locating the researcher/ Q7: Influence of the researcher on the research addressed/ Q8: Adequate representation of participants and their voices/ Q9: Ethical approval by an appropriate body/ Q10: Conclusions flow from the analysis/interpretation of data.

Table 5. Quality Assessment of Randomized Controlled Trials (JBI Critical Appraisal Checklist)

**Study**	**Q1**	**Q2**	**Q3**	**Q4**	**Q5**	**Q6**	**Q7**	**Q8**	**Q9**	**Q10**	**Q11**	**Q12**	**Q13**	**Score**	**Rating**
Smith et al. (2024)	Y	Y	Y	N	N	N	Y	Y	Y	Y	Y	Y	Y	10/13	High

Note: JBI Critical Appraisal Checklist for Randomized Controlled Trials: Q1: Was true randomization used for assignment of participants/Q2: Was allocation to treatment groups concealed?/Q3: Were treatment groups similar at the baseline?/Q4: Were participants blind to treatment assignment?/Q5: Were those delivering treatment blind to assignment?/Q6: Were outcomes assessors blind to treatment assignment?/Q7: Were treatment groups treated identically other than the intervention?/Q8: Was follow-up complete and analyzed by intention to treat?/Q9: Were participants analyzed in the groups to which they were randomized?/Q10: Were outcomes measured in the same

way for treatment groups?/Q11: Were outcomes measured in a reliable way?/Q12: Was appropriate statistical analysis used?/Q13: Was the trial design appropriate for the specific topic?
